# Emergence of unconventional ferroelectric phase in ultrathin Hf_0.5_Zr_0.5_O_2_ films

**DOI:** 10.1126/sciadv.adz8245

**Published:** 2026-05-20

**Authors:** Sangjun Lee, Hyangsook Lee, Hyun Hwi Lee, Han-Koo Lee, Jung-Hwa Kim, Seontae Park, Hyun Jae Lee, Hagyoul Bae, Sanghyun Jo, Musarrat Hasan, Yongho Ha, Bong Jin Kuh, Jinseong Heo, Duk-Hyun Choe, Eunha Lee

**Affiliations:** ^1^ Samsung Advanced Institute of Technology, Suwon-si, Gyeonggi-do 16678, Republic of Korea.; ^2^ Pohang Accelerator Laboratory (PAL), POSTECH, Pohang 37673, Republic of Korea.; ^3^ Department of Electronic Engineering, Jeonbuk National University, Jeonju-si 54896, Republic of Korea.; ^4^ Semiconductor R&D Center, Samsung Electronics, Hwaseong 18448, Republic of Korea.

## Abstract

Unlike perovskite-based ferroelectrics, whose polarization typically diminishes at reduced thickness, doped hafnia films retain robust ferroelectricity down to 1-nanometer thickness. Accurate structural determination is crucial for understanding this behavior, as ferroelectricity in ultrathin hafnia-based films has been attributed to enhanced polar distortion in their crystal structures. However, precise identification is challenging because of structural similarities among various polymorphs. Here, we experimentally identify the emergence of an unconventional ferroelectric Pmn2_1_ orthorhombic phase in 1.5-nanometer-thick Hf_0.5_Zr_0.5_O_2_ films directly grown on silicon substrates. Structural and spectroscopic analyses clearly distinguish the experimentally observed Pmn2_1_ phase from the conventional Pca2_1_ phase typically reported in thicker hafnia films. Furthermore, we find that substantial expansion of the out-of-plane lattice dimension at ultrathin scales drives the stabilization of the Pmn2_1_ structure. The experimental identification of this unconventional phase provides crucial insights into the structural evolution underlying the distinct thickness-dependent ferroelectric properties of ultrathin hafnia films.

## Teaser

Ultrathin hafnia films reveal an unexpected crystal structure, enabling robust electric polarization at atomic-scale thickness.

## INTRODUCTION

The discovery of ferroelectricity in doped hafnia has prompted extensive research to understand its fundamental origin. While first-principles calculations predict several metastable ferroelectric phases in hafnia (*1*, *2*), ferroelectricity is generally attributed to the presence of the Pca2_1_ polar orthorhombic (oIII) phase (*3*–*6*). Epitaxially grown hafnia is an exception, with experimental reports of R3m polar rhombohedral (r) phases in epitaxial Hf_0.5_Zr_0.5_O_2_ (HZO) grown on La_0.7_Sr_0.3_MnO_3_/SrTiO_3_ (*7*), ZnO (*8*), and GaN (*9*) substrates. In contrast, theoretical calculations suggest that the Pmn2_1_ polar orthorhombic (oIV) phase (*1*, *10*–*12*) is responsible for the observed ferroelectricity in epitaxial hafnia rather than the r phase (*10*, *13*). Overall, the structural origin of ferroelectricity in hafnia-based thin films remains unresolved.

Scalable ferroelectrics that can be grown directly on silicon using the mature atomic layer deposition (ALD) process are critical for the development of industrial-scale devices (*5*, *14*). For ALD-grown HZO on silicon, ferroelectricity is primarily attributed to the oIII phase. While there have been prior claims of the unconventional oIV phase in hafnia-based films (*15*–*17*), these reports lacked rigorous structural evidence and remain speculative. To date, the oIII phase remains the only phase consistently established in ALD-grown films (*18*, *19*). However, the intrinsic properties of the oIII phase—such as its large coercive field exceeding 0.8 MV/cm—pose challenges for further performance optimization (*20*). Despite the dominance of the single reported phase, HZO still demonstrates a pronounced thickness-dependent behavior (*5*, *6*, *21*, *22*), suggesting the potential existence of other undiscovered phases.

**Fig. 1.**
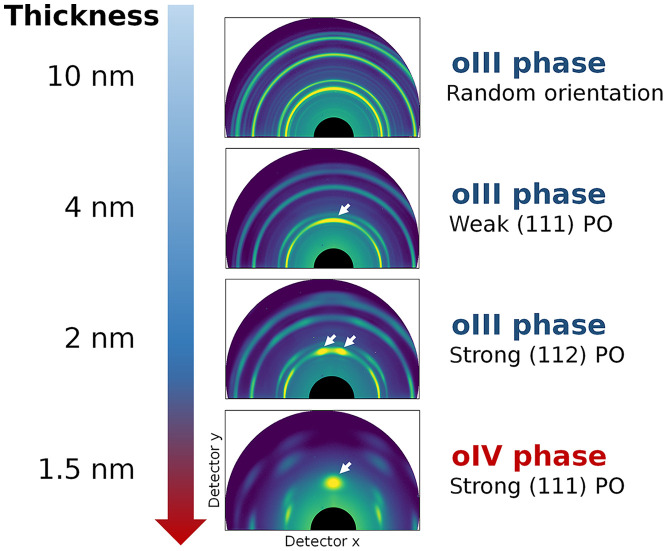
Structural evolution of HZO films as thickness decreases from 10 to 1.5 nm. 2D GIXRD images at various thicknesses reveal changes in preferred orientation (PO), as indicated by the texturing of the (111) peak (white arrow). Our structural analysis indicates the evolution from the oIII phase in thicker films to the oIV phase in the 1.5-nm-thick film. The identification of the oIV phase is based on the detailed structural analysis described in the main text.

Figure 1 depicts the thickness-dependent structural evolution of HZO films grown on silicon. In films with thicknesses greater than 10 nm, the formation of the monoclinic (m) phase is favored, leading to suppressed ferroelectricity. As the film thickness decreases below 10 nm, the oIII phase becomes more favorable than the m phase, leading to ferroelectricity. Films with this thickness range show a randomly oriented polycrystalline structure, with a slight preference for (111) or (001) orientations depending on the fabrication conditions. At a thickness of 2 nm, HZO films exhibit intriguing textures, primarily (112) orientation, retaining the oIII phase (*6*). In the ultrathin limit, only a few experiments have been conducted to date. Unlike conventional ferroelectrics in which depolarizing fields dominate at thicknesses of a few nanometers, recent studies have revealed a marked improvement in ferroelectricity, including reverse size effects (*5*, *21*), and the negative capacitance effect (*23*, *24*) even at a thickness of 1 nm in HZO. However, the underlying physics and precise crystal structures remain unclear, largely because of the experimental challenges of accurately characterizing ultrathin films.

Here, we report the emergence of the Pmn2_1_ polar orthorhombic (oIV) phase in a 1.5-nm-thick HZO film grown on Si by ALD. The crystal structure is thoroughly characterized using x-ray diffraction (XRD) and scanning transmission electron microscopy (STEM), revealing distinctive features that distinguish the oIV phase from the conventional oIII phase. Combined with XRD, oxygen K-edge x-ray absorption spectroscopy (XAS) measurements and simulations across different thicknesses indicate phase transformation from oIII to oIV below a thickness of 2 nm. Density functional theory (DFT) calculations suggest that the expansion of out-of-plane *d*_(111)_ upon reducing thickness makes the oIV phase favorable over other polymorphs. Our study provides precise characterization of the crystal structure at the ultrathin limit, elucidating the structural origin of the intriguing ferroelectric properties observed in ultrathin HZO.

## RESULTS AND DISCUSSION

### Identification of the oIV ferroelectric phase in ultrathin HZO

**Fig. 2.**
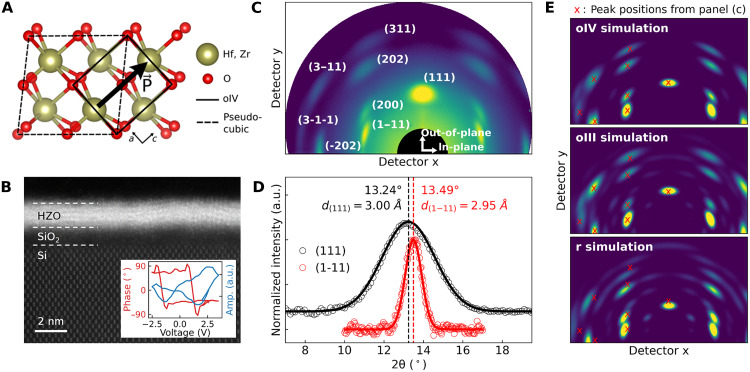
Identification of the oIV phase in 1.5-nm-thick HZO film. (**A**) Crystal structure of oIV phase. The oIV phase unit cell (solid lines) and the pseudo-cubic unit cell (dashed lines) are shown together. The noncentrosymmetric displacement of oxygen atoms induces spontaneous polarization P. (**B**) HAADF-STEM image of a cross section of the 1.5-nm-thick HZO film sample. (Inset) PFM profile showing a ferroelectric hysteresis loop. (**C**) 2D GIXRD image, with peaks indexed using the pseudo-cubic unit cell. (**D**) Non-GI line scans of the (111) and (1–11) peaks. Solid lines are fits using a Gaussian function. Backgrounds are subtracted from the scans, which are then normalized to have the same peak intensities. (**E**) Simulated 2D GIXRD pattern for the oIV, oIII, and r phases with a strong (111) preferred orientation. Overlaid are experimental peak positions (red crosses) obtained from the 2D GIXRD image in (C). a.u., arbitrary units.

We review the crystal structure and ferroelectric properties of the oIV phase of HZO (see fig. S1 for the comparison of the crystal structures of the oIII, oIV, and r phases). Figure 2A shows the crystal structure projected onto the *a*-*c* plane of the oIV unit cell. The centrosymmetry is broken by the displacement of oxygen atoms, resulting in spontaneous polarization along the *c* axis. Solid and dashed lines represent the oIV and pseudo-cubic unit cells, respectively. For consistency, we use the pseudo-cubic unit cell as the basis for Miller indices. Previous theoretical studies estimated a remnant polarization of 56 μC/cm^2^ and a significantly lower switching barrier of 8 meV/atom, compared to 40 meV/atom in the oIII phase (*1*, *11*), suggesting that the oIV phase may exhibit a lower coercive field and be more suitable for low-power device applications.

Using XRD and STEM, we identify the formation of the oIV phase in a 1.5-nm-thick HZO film. Figure 2B shows a high-angle annular dark-field STEM (HAADF-STEM) image of the cross section of the sample, illustrating the direct growth of ultrathin HZO on a Si substrate with an interfacial SiO_2_ layer. The piezoresponse force microscopy (PFM) measurement (inset of Fig. 2B) shows a typical ferroelectric hysteresis loop, confirming the ferroelectric nature of the crystal phase in the film, as in previous studies (*5*, *24*). As discussed earlier, the ferroelectricity of HZO has been attributed to the formation of either the oIII, oIV, or r phase. To determine the specific phase present in the film, we conducted a two-dimensional (2D) grazing incidence (GI) XRD measurement (Fig. 2C). The film exhibits a strong preferred orientation of (111) along the out-of-plane direction, as evidenced by a sharp, localized diffraction intensity distribution. The peak positions of the off-axis planes in the 2D GIXRD pattern, determined by interplanar angles relative to the (111) plane and lattice spacings, enable phase identification through comparison with simulations. Figure 2E compares the experimental 2D GIXRD pattern with simulated patterns of the oIV, oIII, and r phases (see fig. S2 for details of the 2D GIXRD simulations). The major peaks in the experimental pattern align well with those of the oIV and oIII phases. However, we find a significant mismatch with those of the r phase, indicating that the r phase is not present.

Because of structural similarities, the simulated patterns of the oIV and oIII phases closely resemble each other, with the exception of the presence of weak peaks that may be undetectable in the experimental pattern due to limitations in sensitivity. A decisive criterion for distinguishing between the two phases is that *d*_(111)_ is larger than *d*_(1−11)_ in oIV, whereas they are identical in the oIII phase (*10*). To ensure precise lattice spacing determination, we performed XRD measurements in a non-GI geometry (incidence angle of 6.8°), which mitigates undesired refraction effects that cause shifts in the peak positions in GI measurements (*25*–*27*). In Fig. 2D, line scan results show a slight shift of the (1−11) peak relative to the (111) peak, with Gaussian fits showing that *d*_(111)_ (3.00 Å) is larger than *d*_(1−11)_ (2.95 Å). This difference confirms the identification of the oIV phase over the oIII phase. This observation also excludes the tetragonal (t) phase, since in the t phase, the (111) and (1−11) reflections in the pseudocubic notation are symmetry-equivalent and must yield identical *d*-spacings, which is inconsistent with the peak splitting observed in Fig. 2D (see also fig. S3 for exclusion of the m phase). We note that the (111) peak, which lies along the out-of-plane direction, is significantly broadened because the correlation length is limited by the film thickness (1.5 nm). In contrast, the (1–11) peak is tilted by approximately 70° from the out-of-plane, closer to the in-plane direction, where the correlation length is governed by the grain size, which is several nanometers (see fig. S7).

**Fig. 3.**
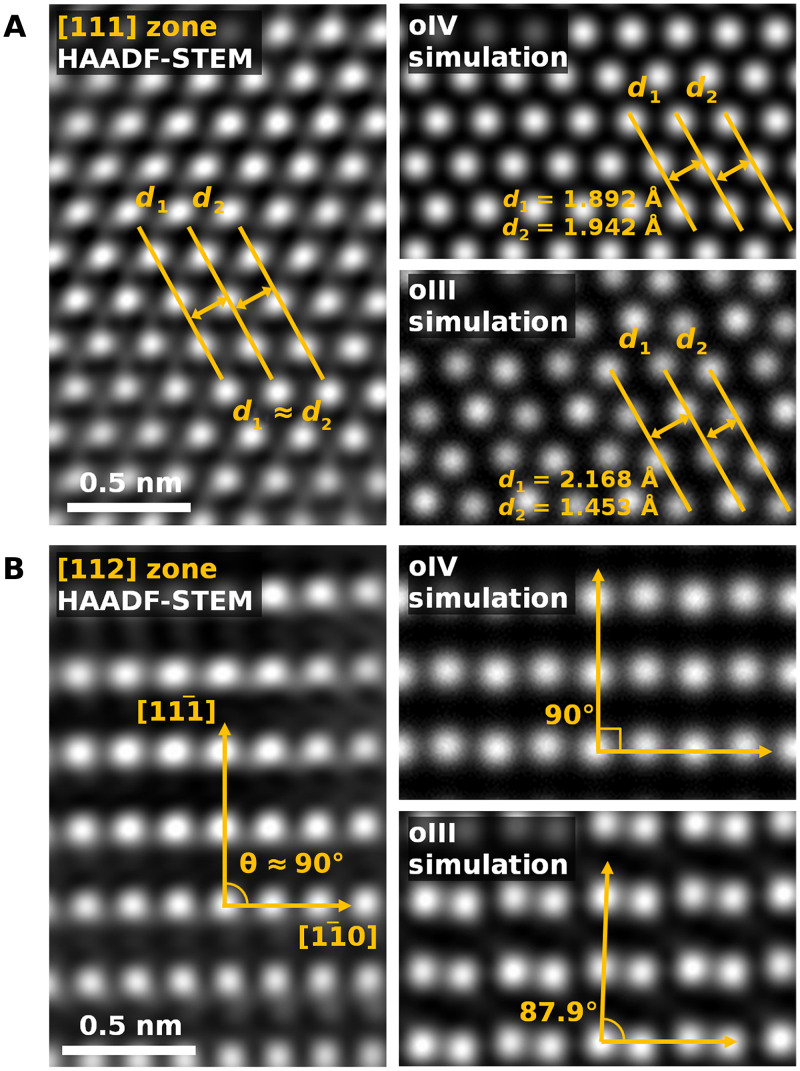
Atomic arrangements of 1.5-nm-thick HZO. (**A**) Plan-view HAADF-STEM image along the [111] zone axis (left) and simulated images of the oIV (right top) and oIII (right bottom) phase projected along the same zone axis. A comparison of the interplanar distances *d*_1_ and *d*_2_ reveals that the experimental image corresponds to the arrangement of the oIV phase. (**B**) Similar comparison along the [112] zone axis. The measured angle between [1$\bar{1}$0] and [11$\bar{1}$] is found to be close to 90°, which aligns with the expected geometry of the oIV phase.

Further evidence of the oIV phase formation is obtained from direct measurements of the local arrangement of Hf/Zr atoms in atomic-resolution STEM images. Figure 3 shows plan-view aberration-corrected HAADF-STEM images of two grains with the [111] and [112] zone axes, indexed using the pseudocubic unit cell. While the polymorphs of HZO exhibit very similar projected atomic arrangements in the pseudocubic framework, a detailed analysis reveals subtle but measurable differences. In the [111] zone axis experimental image (Fig. 3A), the interplanar distances *d*_1_ and *d*_2_ show no discernible difference within the measurement resolution of 0.07 Å. This observation is consistent with the oIV phase simulation, which predicts a 0.05-Å difference. By contrast, the oIII phase is expected to show a 10-fold greater difference. In the [112] zone axis image (Fig. 3B), the angle between the [1$\bar{1}$0] and [11$\bar{1}$] planes is measured to be nearly 90°, which matches the results of the oIV phase simulation and is in contrast to that of the oIII phase (87.9°).

### Thickness-driven phase evolution

**Fig. 4.**
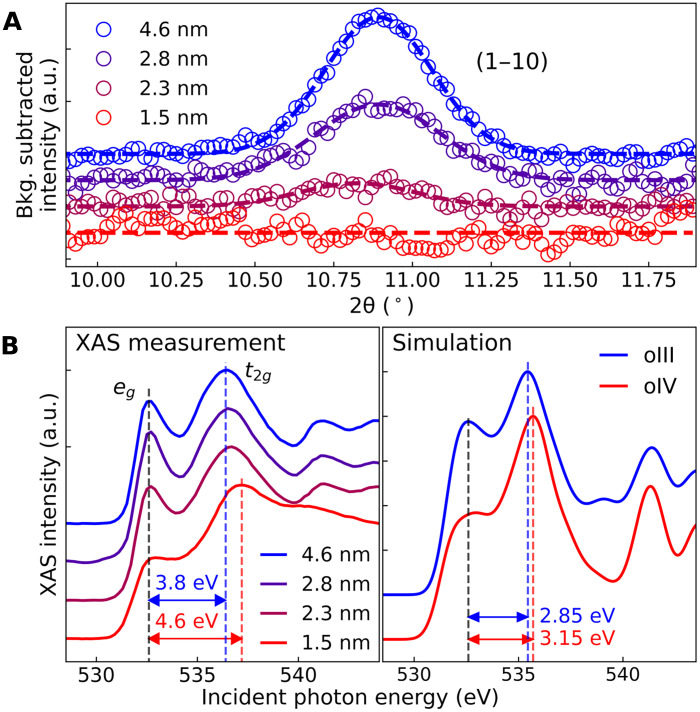
Structural and spectroscopic evidence of phase transformation upon reducing thickness. (**A**) GIXRD line scans of the (1–10) peak. The disappearance of the (1−10) peak at the 1.5-nm thickness indicates structural phase transformation, as the (1−10) peak is a forbidden reflection in the oIV phase. (**B**) Oxygen K-edge XAS measurements (left) showing changes in the spectral line shape and *e_g_*-*t_2g_* splitting. Simulated spectra of the oIII and oIV phases (right) reveal similar changes in the line shape and the energy splitting.

Upon confirming the oIV phase in the 1.5-nm-thick film, we investigated structural changes with varying thicknesses. To this end, we performed GIXRD measurements on samples with thicknesses of 1.5, 2.3, 2.8, and 4.6 nm. At the thickest end, the 4.6-nm-thick sample predominantly exhibited the oIII phase, with a minor presence of the m phase (see fig. S5). The characteristic feature distinguishing the oIV phase from the oIII phase is the presence of the (110) reflection (*10*), which is forbidden in the oIV phase but allowed in the oIII phase. To track phase evolution, we measured the (1−10) peak, which is symmetry-equivalent to (110), in samples with different thicknesses. Line scans were taken from the 2D GIXRD images along the in-plane direction, considering the preferred orientation of the samples (see fig. S6). Figure 4A shows that the (1−10) peak persists down to a thickness of 2.3 nm, albeit with diminished intensity, and disappears at 1.5 nm. This indicates that the most stable form of crystal phase transforms from oIII to oIV as the film thickness is reduced to 1.5 nm.

The change in the crystal structure is also evident in the oxygen K-edge XAS spectra (Fig. 4B). The two primary features at approximately 532.5 and 536.5 eV correspond to excitations to *e*_g_ and *t*_*2*g_ orbitals, originating from the crystal field splitting in fluorite structures (*5*, *28*, *29*). The energy difference between these features, denoted as Δ*E*, represents the degree of tetrahedral distortion in the crystal structure. In the 4.6-nm-thick film where the oIII phase is formed, Δ*E* is 3.8 eV, which is comparable to previously reported results (*5*). We observe that Δ*E* increases as the film thickness decreases, reaching up to 4.6 eV in the 1.5-nm-thick film. This increase is attributed to greater distortions in the crystal structure induced by the formation of the oIV phase. Furthermore, spectral weight shifts from the *e_g_* band to the *t_2g_* band with decreasing thickness. DFT calculations simulated the XAS profiles for the oIII and oIV phases, showing increased spectral weights at the *t_2g_* band and a higher Δ*E* for the oIV phase (Fig. 4B). These results closely align with the experimental observations, although the Δ*E* values are slightly underestimated likely because of the limitations of DFT (*30*). This qualitative agreement corroborates the observed structural change.

### Mechanism of oIV stabilization

It is indeed intriguing that the emergence of the oIV phase is solely triggered by altering the film thickness, without any other modifications to growth conditions or adjacent layers. The established mechanism for ferroelectric phase formation in HZO typically involves the formation of a high-temperature nonpolar t phase during the postannealing process, which then transitions to a polar phase. Several theoretical studies have shown that both the oIII and oIV are viable phases that can be stabilized from the t phase, each driven by distinct soft phonon modes, and are energetically very close (*1*, *31*). As discussed below, our observations suggest that a specific change in the microstructure of the 1.5-nm-thick film, driven by a thickness-dependent effect, makes the transition from the t phase to the oIV phase energetically more favorable than to the oIII phase.

**Fig. 5.**
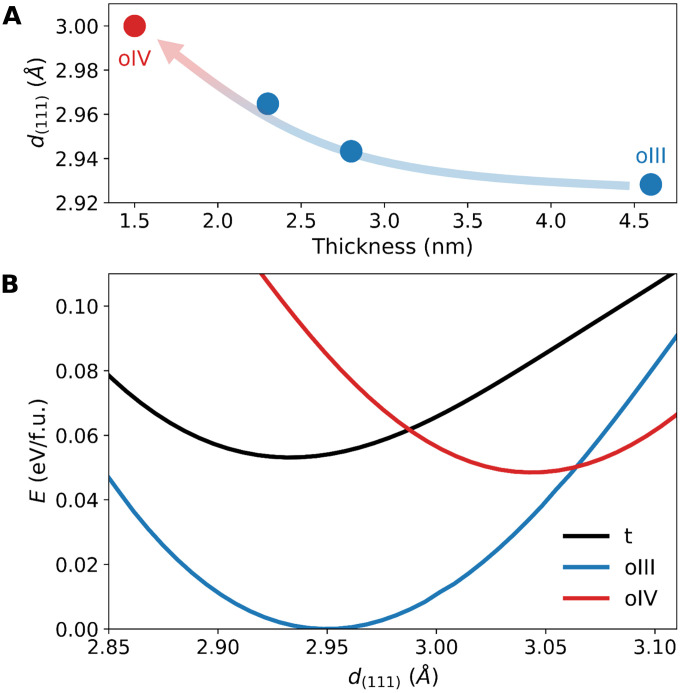
Stabilization of the oIV phase through out-of-plane lattice expansion. (**A**) Variation of out-of-plane d_(111)_ with thickness, illustrating lattice expansion as thickness decreases. (**B**) Total energy of the t, oIII, and oIV phases as a function of *d*_(111)_. Above certain values of *d*_(111)_, the oIV phase becomes more stable than the t and oIII phases. The energy minimum of the oIII phase is selected as the zero-energy reference.

From a structural perspective, the key difference observed among the samples is the expansion of the out-of-plane lattice with decreasing thickness. Figure 5A shows the variation in the out-of-plane *d*_(111)_, the *d*-spacing of the (111) plane aligned along the out-of-plane direction. In films thicker than 2 nm, where the oIII phase is dominant, *d*_(111)_ expands from 2.93 to 2.97 Å as the thickness decreases from 4.6 to 2.3 nm. When the thickness reduces to 1.5 nm, corresponding to the emergence of the oIV phase, *d*_(111)_ further expands to 3.00 Å. There have been similar reports of the out-of-plane *d*_(111)_ expansion in Hf_0.8_Zr_0.2_O_2_ and ZrO_2_ grown on Si (*5*, *21*), suggesting an inherent thickness effect in ultrathin hafnia and zirconia films (*32*).

To examine the influence of lattice expansion on the stability of each phase, we compared the relative total energies as a function of *d*_(111)_ using DFT calculations (Fig. 5B). At a *d*_(111)_ of approximately 2.95 Å, the oIII phase is most stable, consistent with our experimental observations. However, as the lattice expands, the oIII phase becomes relatively unstable, while the energy of the oIV phase decreases significantly. Once *d*_(111)_ exceeds 3.06 Å, the oIV phase becomes the most energetically favorable. Although a quantitative comparison of the critical value of *d*_(111)_ with the experimental value is limited, our findings suggest that out-of-plane lattice expansion can promote oIV phase formation.

Microstructural changes and surface/interface energies can also play crucial roles, especially given the ultrathin nature of the samples. For instance, we observe a significant reduction in grain size with decreasing thickness (see fig. S7). This reduction in grain size may contribute to the lattice expansion, as previously observed in various metal oxides, including hafnia thin films (*33*) and nanocrystals (*34*–*36*), although a definitive explanation remains elusive (*37*). The strong preferred orientation in the microstructure could also make HZO more susceptible to phase transition, as Qi *et al.* (*10*) have shown that the (111) orientation, which we also observe in our 1.5-nm sample, promotes oIV phase formation under slight shear strain.

In summary, we have revealed the formation of the Pmn2_1_ oIV phase in a 1.5-nm-thick HZO film, overcoming experimental challenges through a combination of XRD, TEM, XAS, and DFT calculations. Our results provide compelling evidence that the oIV phase is responsible for the enhanced ferroelectricity observed in ultrathin HZO. A comparison with thicker films, which exhibit the conventional Pca2_1_ oIII phase, highlights the unique structural and spectroscopic characteristics of the oIV phase. Theoretical calculations further demonstrate that the expansion of the out-of-plane lattice in the 1.5-nm-thick film plays a critical role in stabilizing the oIV phase.

Our study positions the oIV phase as a promising alternative to the oIII phase, whose high coercive field imposes limitations on energy-efficient ferroelectric device applications. The high remnant polarization and potential low coercive field, combined with the scalability and ALD compatibility of HZO, make the oIV phase highly attractive for industrial applications. Furthermore, our findings open possibilities for synthesizing the oIV phase in thicker films, which could substantially broaden its potential applications.

## MATERIALS AND METHODS

### HZO film synthesis

The HZO films were deposited on Si (100) substrates using the ALD process at 200°C using tetrakis-(ethylmethylamido)hafnium (99.99%), tetrakis-(ethylmethylamido)zirconium (99.99%), and H_2_O precursors. Alternating cycles for HfO_2_ and ZrO_2_ depositions were performed to achieve the HZO film. Postdeposition annealing was carried out using laser annealing at 1100°C. We note that the oIV phase was also observed in a 1.5-nm film crystallized by conventional rapid thermal annealing at 700°C (see fig. S9), confirming that its formation is not dependent on the specific annealing method.

### TEM sample preparation

Specimens for cross-sectional TEM were prepared using a focused-ion beam system (Thermo Fisher Scientific Helios F-1). Specimens for plane-view TEM were prepared by capping with an amorphous carbon protective film after removing the Mo top layer using a chemical etching process. Subsequently, dimpling and Ar ion-milling were applied.

### STEM imaging

HAADF-STEM analyses were performed at an accelerating voltage of 300 kV using the aberration-corrected Thermo Fisher Scientific Titan3 G2 60-300 system. The HAADF-STEM images were acquired at collection semiangles of 50 to 200 mrad. Atomic-resolution HAADF-STEM image simulations were performed using the HREM software, with a probe size of 1 Å.

### Plan-view TEM-PED

The precession electron diffraction (PED) experiment was carried out at 200 kV with the probe diameter of approximately 2 nm using the NanoMegas system equipped with the Thermo Fisher Scientific Osiris TEM. On the basis of the acquired electron diffraction patterns, the crystal structures were indexed through template-matching using the ASTAR software. The PED mapping reveals a dominant formation of oIV phase in the film (see fig. S8).

### Synchrotron x-ray measurements

XRD measurements were performed at the 5A undulator beamline at Pohang Light Source II (PLS-II, Pohang, Korea). An x-ray energy of 17.9 keV (λ = 0.6926 Å) was used for all XRD measurements and an incidence angle of α = 0.10° was used for GI measurements. The 2D XRD patterns were collected by a Mar345 image plate detector. Oxygen K-edge XAS measurements were performed at the 10A2 beamline at PLS-II. The spectra were obtained in total electron yield mode.

### PFM measurement

PFM hysteresis loops were measured using commercial atomic force microscopy systems embedded with a LabVIEW/MATLAB-based band excitation controller and data acquisition systems (National Instruments NI-PXIe 5122/5412). The PFM hysteresis loop data were measured in the range of −2.5 to 3 V by applying a gradually increasing alternating-current modulation voltage of 0.8 V at 300 to 400 kHz.

### DFT calculations

DFT calculations were performed using the Perdew-Burke-Ernzerhof functional for solids (*38*) for the exchange-correlation potential and the projector augmented wave potentials (*39*), as implemented in the Vienna Ab initio Simulation Package code (*40*). The wave functions were expanded as plane waves up to an energy cutoff of 800 eV, and the force tolerance for structure optimization was set as 0.01 eV/Å. We used Γ-centered k points generated by 6 by 6 by 6 Monkhorst-Pack meshes for a pseudo-cubic unit cell and accordingly adjusted them for the supercell calculations. To model the HZO structure, we selected the most stable Hf/Zr configurations for each phase among all possible configurations within pseudo-cubic unit cells. XAS calculations were performed on the basis of supercell core-hole methods using 2 by 2 by 2 supercells, where an increase in the supercell size negligibly affected the spectra.

## Supplementary Materials

### This PDF file includes:

Figs. S1 to S10 References
