# Supplementary material for: Emergence of unconventional ferroelectric phase in ultrathin Hf0.5Zr0.5O2 films
Source: Sci Adv. 2026 May 20;12(21):eadz8245. doi: 10.1126/sciadv.adz8245 (PMC13189120; doi:10.1126/sciadv.adz8245)
Supplement: Supplementary file 1 — Figs. S1 to S10 References [file sciadv.adz8245_sm.pdf]

Science Advances
AAAS

Supplementary Materials for

Emergence of unconventional ferroelectric phase in ultrathin $Hf_{0.5}Zr_{0.5}O_2$ films

Sangjun Lee *et al.*

Corresponding author: Duk-Hyun Choe, dukhyun.choe@samsung.com; Eunha Lee, eunhayo.lee@samsung.com

Sci. Adv. **12**, eadz8245 (2026)
DOI: 10.1126/sciadv.adz8245

This PDF file includes:

Figs. S1 to S10
References

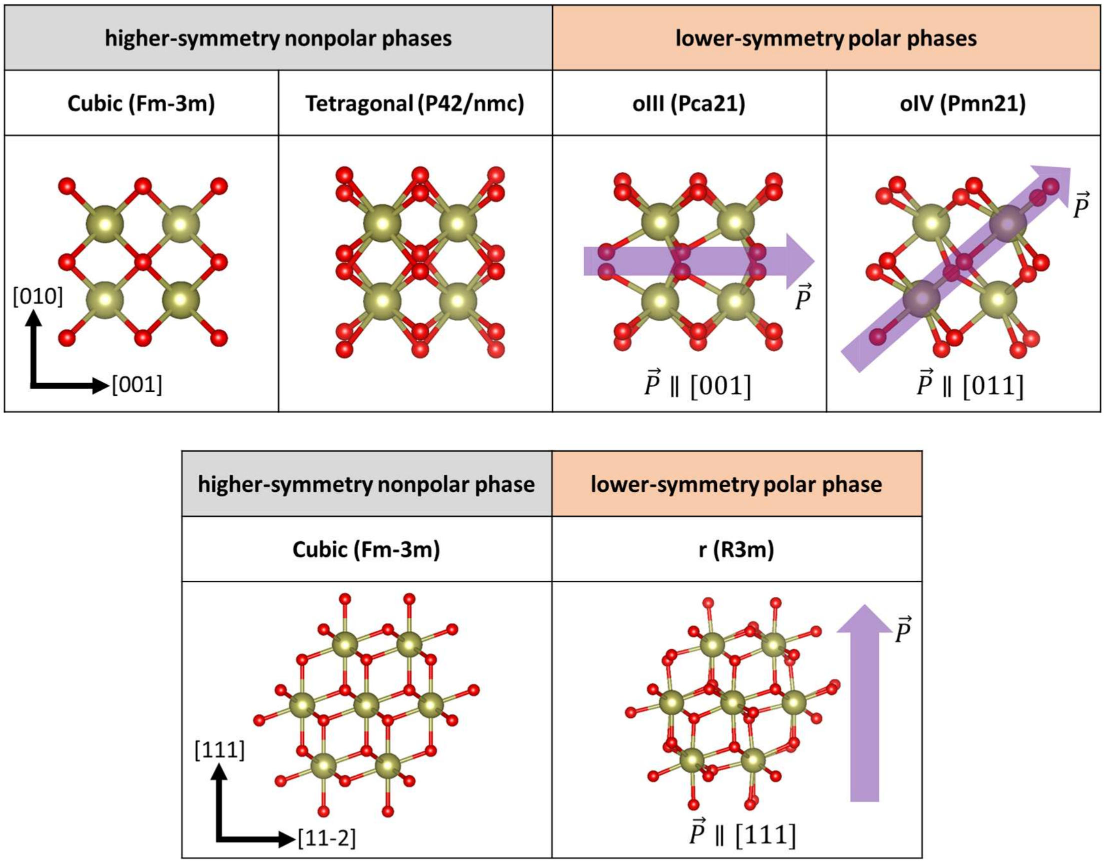

Fig. S1. Comparison of ferroelectric and nonpolar reference crystal structures. Comparison of the crystal structures of the oIII, oIV, and r ferroelectric phases (right) with their corresponding higher-symmetry nonpolar reference phases (left). The figure illustrates the distinct atomic distortions that give rise to spontaneous polarization in each case. Purple arrows indicate the direction of polarization for each polar phase.

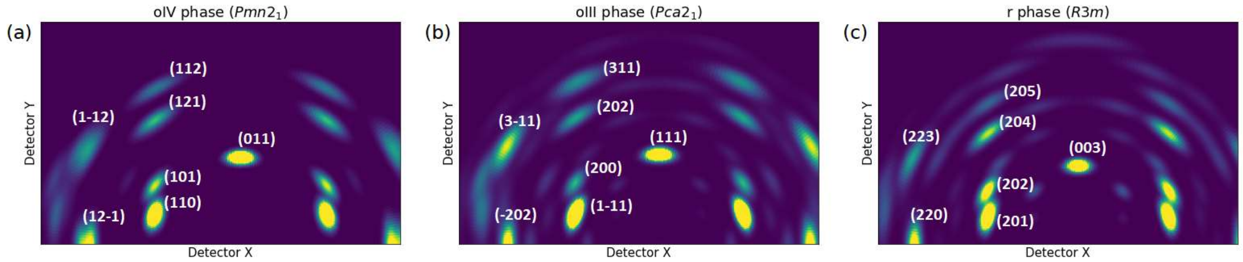

Fig. S2. Simulated 2D GIXRD patterns of candidate ferroelectric phases. 2D GIXRD pattern simulations for the (a) oIV, (b) oIII, and (c) r phases. The unit cell parameters for the oIV and oIII phases are taken from Qi et. al. (10), and those for the r phase from Wei et al. (7). For each case, a strong preferred orientation along the out-of-plane direction and random orientation in the in-plane direction are assumed. The preferred orientation direction is selected to produce the closest match to the experimental pattern. Major reflections are indexed based on the unit cell of each phase. Note that the presented patterns simulate images collected by a flat area detector in a grazing incidence geometry. While they are similar to reciprocal space maps, they are not exact due to the curved surface of the Ewald sphere (41).

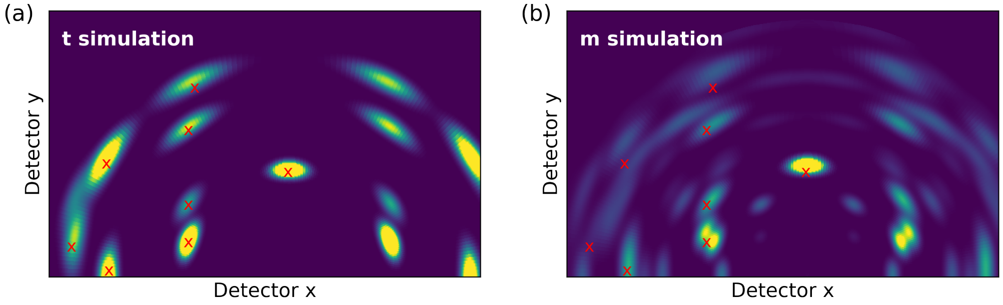

Fig. S3. Exclusion of tetragonal and monoclinic phases. 2D GIXRD pattern simulations for the (a) tetragonal (t) and (b) monoclinic (m) phases. Overlaid are experimental peak positions (red crosses) obtained from the 2D GIXRD image in Fig. 2(c). The t-phase simulation shows partial agreement with the experimental peak positions due to structural similarities with the o-phases. However, as discussed in the main text, the difference between $d_{(111)}$ and $d_{(1-11)}$ observed experimentally allows the t-phase to be excluded. The simulated m-phase pattern is distinct from the experimental peaks, further confirming that the m-phase is not present.

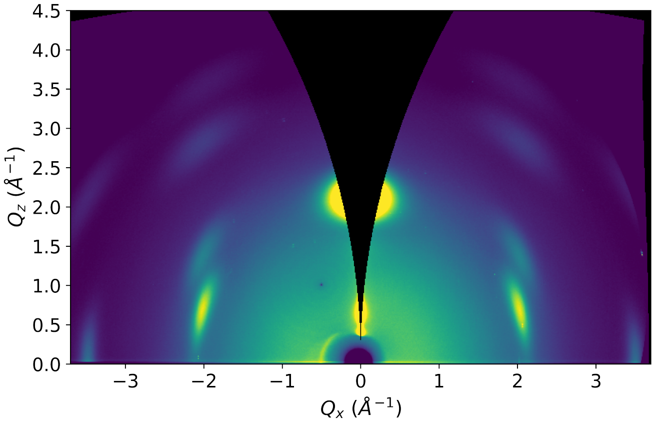

Fig. S4. Reciprocal space representation of 2D GIXRD data. Reciprocal space representation (Q_x–Q_z map) of the experimental 2D GIXRD image shown in Fig. 2(c). Q_x corresponds to the in-plane reciprocal space direction and Q_z to the out-of-plane direction. The conversion provides a physically interpretable view of the diffraction geometry, while the curved Ewald sphere in GIXRD leads to a blind spot along Q_z (41).

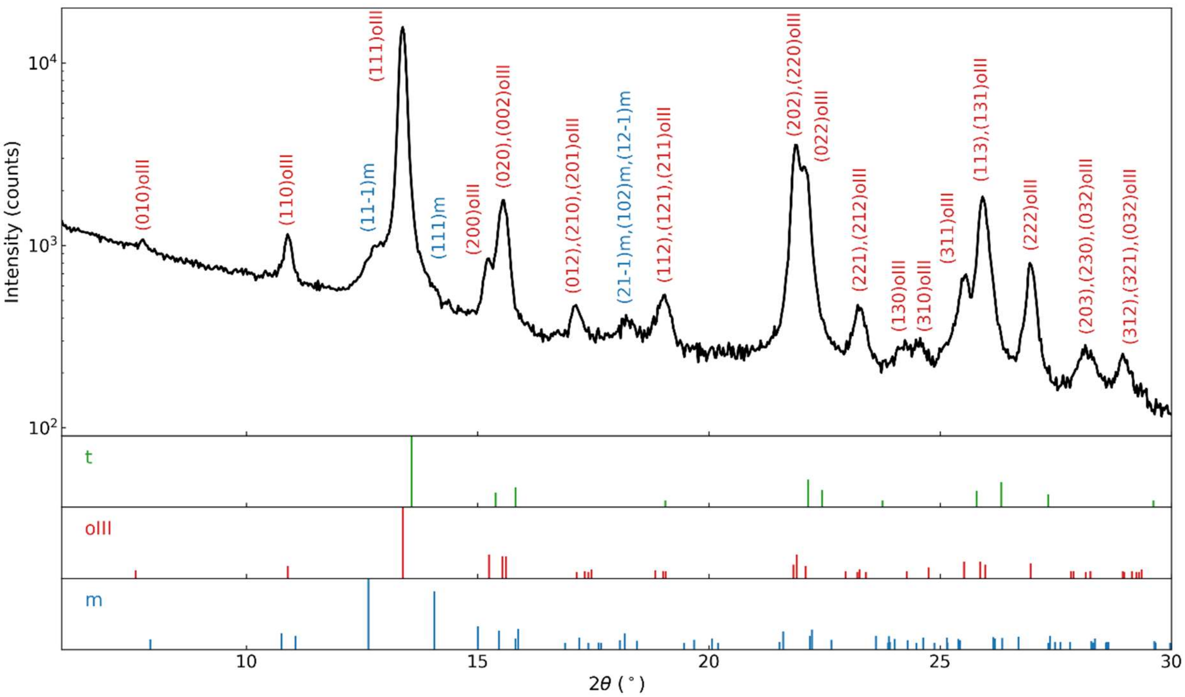

Fig. S5. In-plane GIXRD pattern of the 4.6-nm-thick HZO film. In-plane GIXRD pattern of the 4.6-nm-thick HZO film (top panel) and the reference powder diffraction patterns of the t, oIII, and m phases (bottom panels). The majority of the peaks match well with the diffraction pattern of the oIII phase, and a few weak m phase peaks are also observed.

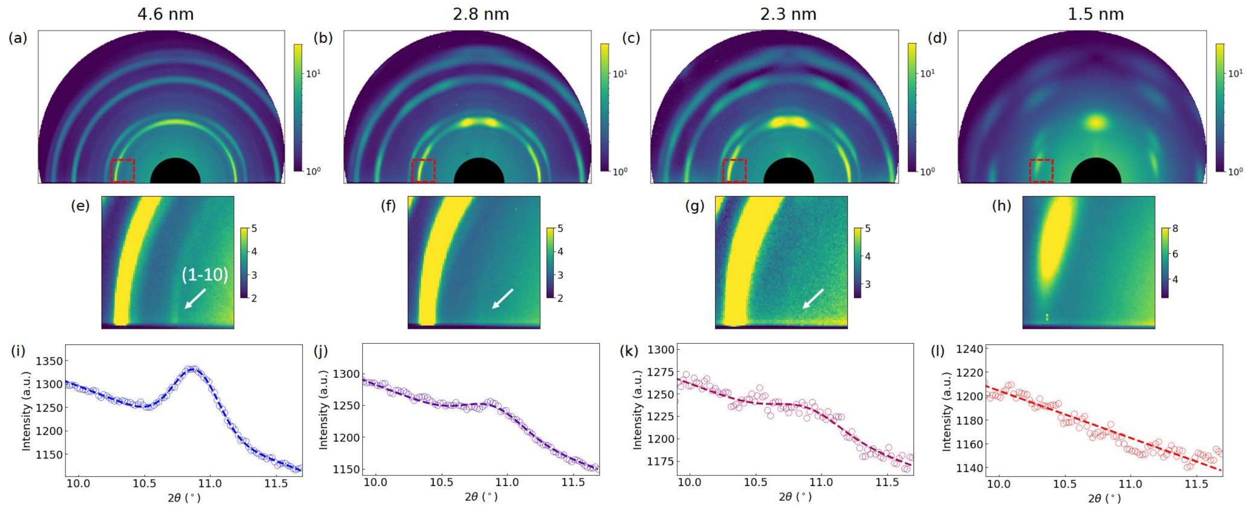

Fig. S6. Thickness-dependent evolution of the (1−10) reflection. (a,b,c,d) 2D GIXRD images for the samples with thicknesses of 4.6, 2.8, 2.3, and 1.5 nm. The 4.6-nm-thick sample shows a weak (111) preferred orientation along the out-of-plane direction. The 2.8-nm and 2.3-nm-thick samples exhibit a strong (112) preferred orientation, while the 1.5-nm-thick sample shows a strong (111) preferred orientation. The (1−10) is perpendicular to both (111) and (112), thus appearing along the in-plane direction in all samples. (e,f,g,h) Enlarged view of the in-plane region where (1−10) reflection resides (region within the red dashed lines in (a,b,c,d)). The white arrow indicates the (1−10) peak. (i,j,k,l) The line scans of the (1−10) reflection taken from the 2D GIXRD images. The dashed lines are the Gaussian fits with linear backgrounds.

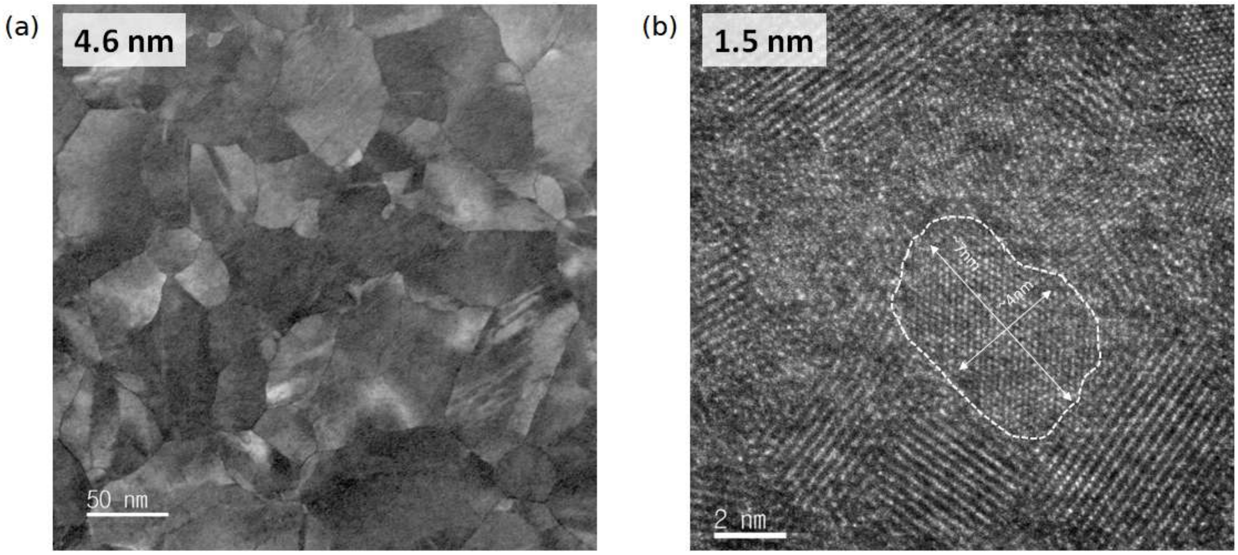

Fig. S7. Grain size reduction with decreasing thickness. Plan-view TEM images of (a) 4.6-nm-thick and (b) 1.5-nm-thick HZO films. At a thickness of 4.6 nm, the typical grain size exceeds 50 nm. As the thickness decreases to 1.5 nm, the grain size significantly reduces to less than 10 nm.

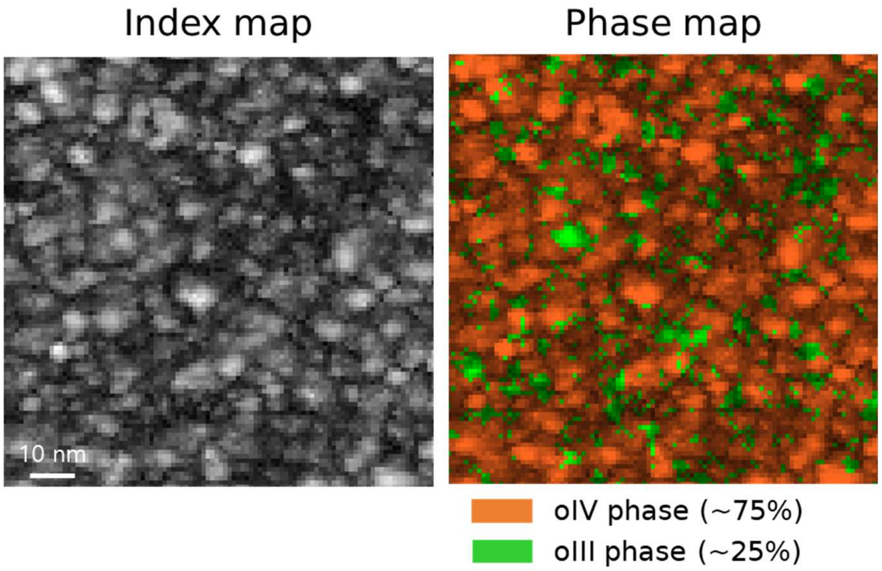

Fig. S8. Precession electron diffraction (PED) mapping of the 1.5-nm HZO film. PED analysis reveals the spatial distribution of oIII and oIV domains in the film. The phase map shows that the oIV phase is dominant, occupying approximately 75% of the measured area, while the remaining ~25% corresponds to the oIII phase. No clear evidence of amorphous regions was observed.

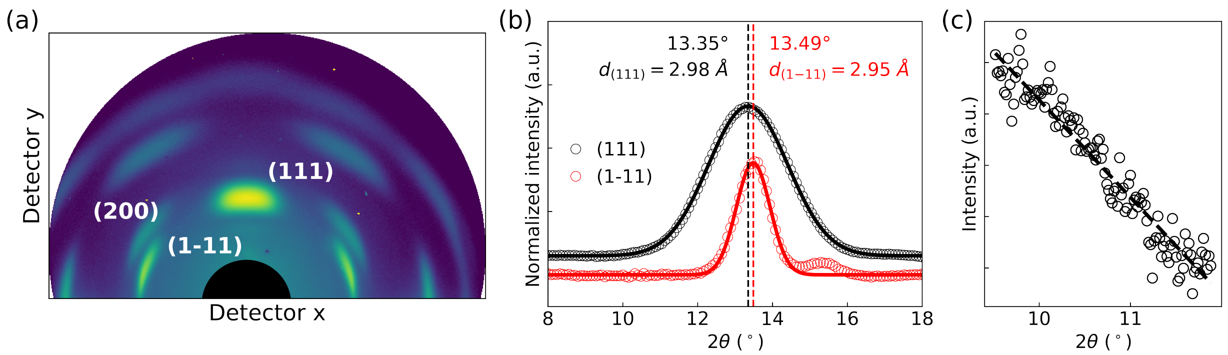

Fig. S9. Structural characterization of the RTA-annealed 1.5-nm HZO film. (a) 2D GIXRD image of the RTA-annealed sample, showing a similar diffraction pattern to the laser-annealed sample (Fig. 2(c)) but with slightly weaker preferred orientation, as indicated by broader peak distributions. (b) Comparison of the (111) and (1−11) peak positions, showing that $d_{(111)} \neq d_{(1-11)}$, consistent with the laser-annealed sample (Fig. 2(d)). This difference serves as a key fingerprint of the oIV phase. Note that, while the non-GI measurement was used for the laser-annealed sample in the main text, this result was obtained from a GIXRD geometry. The refraction effect in GIXRD shifts the (111) peak to higher angles, implying that the true $d_{(111)}$ is slightly larger than shown—further increasing the difference between $d_{(111)}$ and $d_{(1-11)}$. (c) Line scan around the (1–10) position, showing no observable peak, consistent with the absence of the (1–10) reflection in the laser-annealed sample (Fig. 4(a)), confirming the oIV phase formation.

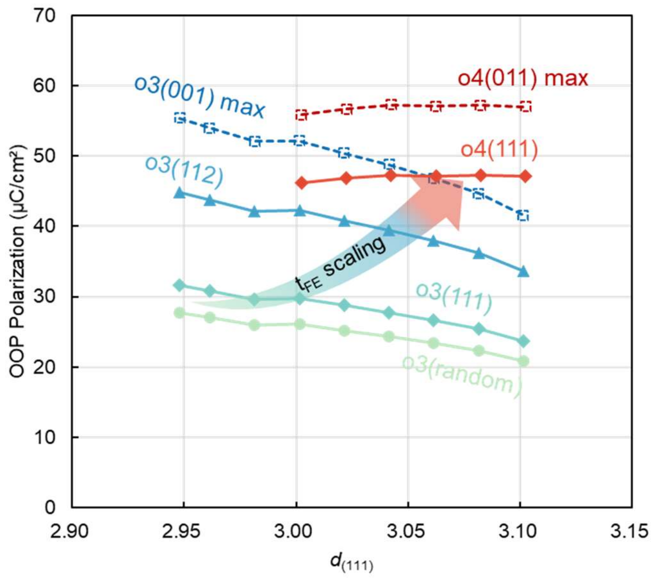

Fig. S10. Orientation-dependent polarization vs. lattice expansion. Calculated out-of-plane (OOP) polarization as a function $d_{(111)}$ for different structural phases (oIII and oIV) and preferred orientations. For the oIII phase, the OOP polarization reaches 50% of its maximum value for completely random orientations, 57% for (111)-textured films, and 82% for (112)-oriented films. In the case of the oIV phase, the (111) orientation yields 83% of the maximum polarization. Combined with the evolution of $d_{(111)}$ with decreasing thickness, it becomes evident that polarization increases with decreasing thickness.

REFERENCES

1. T. D. Huan, V. Sharma, G. A. Rossetti, R. Ramprasad, Pathways towards ferroelectricity in hafnia. *Phys. Rev. B* **90**, 064111 (2014).

2. R. Batra, T. D. Huan, J. L. Jones, G. J. Rossetti, R. Ramprasad, Factors favoring ferroelectricity in hafnia: A first-principles computational study. *J. Phys. Chem. C* **121**, 4139–4145 (2017).

3. T. S. Böscke, J. Müller, D. Bräuhaus, U. Schröder, U. Böttger, Ferroelectricity in hafnium oxide thin films. *Appl. Phys. Lett.* **99,** 102903 (2011).

4. X. Sang, E. D. Grimley, T. Schenk, U. Schroeder, J. M. LeBeau, On the structural origins of ferroelectricity in HfO_2 thin films. *Appl. Phys. Lett.* **106**, 162905 (2015).

5. S. S. Cheema, D. Kwon, N. Shanker, R. dos Reis, S.-L. Hsu, J. Xiao, H. Zhang, R. Wagner, A. Datar, M. R. McCarter, C. R. Serrao, A. K. Yadav, G. Karbasian, C.-H. Hsu, A. J. Tan, L.-C. Wang, V. Thakare, X. Zhang, A. Mehta, E. Karapetrova, R. V. Chopdekar, P. Shafer, E. Arenholz, C. Hu, R. Proksch, R. Ramesh, J. Ciston, S. Salahuddin, Enhanced ferroelectricity in ultrathin films grown directly on silicon. *Nature* **580**, 478–482 (2020).

6. H. Lee, D.-H. Choe, S. Jo, J.-H. Kim, H. H. Lee, H.-J. Shin, Y. Park, S. Kang, Y. Cho, S. Park, T. Moon, D. Eom, M. Leem, Y. Kim, J. Heo, E. Lee, H. Kim, Unveiling the origin of robust ferroelectricity in sub-2 nm hafnium zirconium oxide films. *ACS Appl. Mater. Interfaces* **13**, 36499–36506 (2021).

7. Y. Wei, P. Nukala, M. Salverda, S. Matzen, H. J. Zhao, J. Momand, A. S. Everhardt, G. Agnus, G. R. Blake, P. Lecoeur, B. J. Kooi, J. Íñiguez, B. Dkhil, B. Noheda, A rhombohedral ferroelectric phase in epitaxially strained $Hf_{0.5}Zr_{0.5}O_2$ thin films. *Nat. Mater.* **17**, 1095–1100 (2018).

8. M. Zheng, Z. Yin, Y. Cheng, X. Zhang, J. Wu, J. Qi, Stabilization of thick, rhombohedral $Hf_{0.5}Zr_{0.5}O_2$ epilayer on c-plane ZnO. *Appl. Phys. Lett.* **119**, 172904 (2021).

9. L. Bégon-Lours, M. Mulder, P. Nukala, S. de Graaf, Y. A. Birkhölzer, B. Kooi, B. Noheda, G. Koster, G. Rijnders, Stabilization of phase-pure rhombohedral $HfZrO_4$ in pulsed laser deposited thin films. *Phys. Rev. Mater.* **4**, 043401 (2020).

10. Y. Qi, S. Singh, C. Lau, F.-T. Huang, X. Xu, F. J. Walker, C. H. Ahn, S.-W. Cheong, K. M. Rabe, Stabilization of competing ferroelectric phases of HfO_2 under epitaxial strain. *Phys. Rev. Lett.* **125**, 257603 (2020).

11. Y. Qi, K. M. Rabe, Phase competition in HfO_2 with applied electric field from first principles. *Phys. Rev. B* **102**, 214108 (2020).

12. Y.-W. Chen, C. W. Liu, Effects of shear strain on HZO ferroelectric orthorhombic phases. *Appl. Phys. Lett.* **123**, 112901 (2023)

13. Q. Hu, S. Lv, C. Xue, H. Tsai, T. Cao, Z. Xu, G. Teobaldi, L.-M. Liu, Phase stability and phase transition pathways in the rhombohedral phase of HfO_2. *J. Phys. Chem. Lett.* **15**, 9319–9325 (2024).

14. T. Mikolajick, S. Slesazeck, H. Mulaosmanovic, M. H. Park, S. Fichtner, P. D. Lomenzo, M. Hoffmann, U. Schroeder, Next generation ferroelectric materials for semiconductor process integration and their applications. *J. Appl. Phys.* **129**, 100901 (2021).

15. T. V. Perevalov, V. A. Gritsenko, A. K. Gutakovskii, I. P. Prosvirin, Structure of $Hf_{0.9}La_{0.1}O_2$ ferroelectric films obtained by the atomic layer deposition. *JETP Lett.* **109**, 116–120 (2019).

16. V. Popov, V. Antonov, M. Ilnitsky, I. Tyschenko, V. Vdovin, A. Miakonkikh, K. Rudenko, Ferroelectric properties of SOS and SOI pseudo-MOSFETs with HfO_2 interlayers. *Solid-State Electron.* **159**, 63–70 (2019).

17. V. Popov, M. Ilnitsky, V. Antonov, V. Vdovin, I. Tyschenko, A. Miakonkikh, K. Rudenko, Ferroelectric properties of HfO2 interlayers in SOI and SOS pseudo-MOSFETs. *2018 Joint International EUROSOI Workshop and International Conference on Ultimate Integration on Silicon (EUROSOI-ULIS)* 1–4 (2018)

18. M. H. Park, Y. H. Lee, H. J. Kim, Y. J. Kim, T. Moon, K. D. Kim, J. Müller, A. Kersch, U. Schroeder, T. Mikolajick, C. S. Hwang, Ferroelectricity and antiferroelectricity of doped thin HfO2-based films. *Adv. Mater.* **27**, 1811 (2015), 1831.

19. U. Schroeder, M. H. Park, T. Mikolajick, C. S. Hwang, The fundamentals and applications of ferroelectric HfO_2. *Nat. Rev. Mater.* **7**, 653 (2022), 669.

20. T. Mikolajick, S. Slesazeck, M. H. Park, U. Schroeder, Ferroelectric hafnium oxide for ferroelectric random-access memories and ferroelectric field-effect transistors. *MRS Bullet.* **43**, 340 (2018), 346.

21. S. S. Cheema, N. Shanker, S.-L. Hsu, Y. Rho, C.-H. Hsu, V. A. Stoica, Z. Zhang, J. W. Freeland, P. Shafer, C. P. Grigoropoulos, J. Ciston, S. Salahuddin, Emergent ferroelectricity in subnanometer binary oxide films on silicon. *Science* **376**, 648 (2022), 652.

22. T. Mimura, T. Shimizu, O. Sakata, H. Funakubo, Thickness dependence of phase stability in epitaxial $(Hf_xZr_{1-x})O_2$ films. *Phys. Rev. Mater.* **5**, 114407 (2021).

23. S. S. Cheema, N. Shanker, L.-C. Wang, C.-H. Hsu, S.-L. Hsu, Y.-H. Liao, M. San Jose, J. Gomez, W. Chakraborty, W. Li, J.-H. Bae, S. K. Volkman, D. Kwon, Y. Rho, G. Pinelli, R. Rastogi, D. Pipitone, C. Stull, M. Cook, B. Tyrrell, V. A. Stoica, Z. Zhang, J. W. Freeland, C. J. Tassone, A. Mehta, G. Saheli, D. Thompson, D. I. Suh, W.-T. Koo, K.-J. Nam, D. J. Jung, W.-B. Song, C.-H. Lin, S. Nam, J. Heo, N. Parihar, C. P. Grigoropoulos, P. Shafer, P. Fay, R. Ramesh, S. Mahapatra, J. Ciston, S. Datta, M. Mohamed, C. Hu, S. Salahuddin, Ultrathin ferroic HfO_2–ZrO_2 superlattice gate stack for advanced transistors. *Nature* **604**, 65 (2022), 71.

24. S. Jo, H. Lee, D.-H. Choe, J.-H. Kim, Y. S. Lee, O. Kwon, S. Nam, Y. Park, K. Kim, B. G. Chae, S. Kim, S. Kang, T. Moon, H. Bae, J. Y. Won, D.-J. Yun, M. Jeong, H. H. Lee, Y. Cho, K.-H. Lee, H. J. Lee, S. Lee, K.-J. Nam, D. Jung, B. J. Kuh, D. Ha, Y. Kim, S. Park, Y. Kim, E. Lee, J. Heo, Negative differential capacitance in ultrathin ferroelectric hafnia. *Nat. Electron.* **6**, 390 (2023), 397.

25. M. F. Toney, S. Brennan, Observation of the effect of refraction on x rays diffracted in a grazing-incidence asymmetric Bragg geometry. *Phys. Rev. B* **39**, 7963 (1989), 7966.

26. G. Lim, W. Parrish, C. Ortiz, M. Bellotto, M. Hart, Grazing incidence synchrotron x-ray diffraction method for analyzing thin films. *J. Mater. Res.* **2**, 471 (1987), 477.

27. R. Resel, M. Bainschab, A. Pichler, T. Dingemans, C. Simbrunner, J. Stangl, I. Salzmann, Multiple scattering in grazing-incidence x-ray diffraction: Impact on lattice-constant determination in thin films. *J. Synchrotron Radiat.* **23**, 729 (2016), 734.

28. F. de Groot, A. Kotani, *Core Level Spectroscopy of Solids* (CRC Press., 2008).

29. F. Frati, M. O. J. Y. Hunault, F. M. F. de Groot, Oxygen K-edge x-ray absorption spectra. *Chem. Rev.* **120**, 4056 (2020), 4110.

30. J. P. Perdew, M. Levy, Physical content of the exact Kohn-Sham orbital energies: Band gaps and derivative discontinuities. *Phys. Rev. Lett.* **51**, 1884 (1983), 1887.

31. A. Raeliarijaona, R. E. Cohen, Hafnia HfO_2 is a proper ferroelectric. *Phys. Rev. B* **108**, 094109 (2023).

32. T. Zhu, S. Liu, Origin of reverse size effect in ferroelectric hafnia thin films. arXiv. arXiv:2509.12952 [cond-mat.mtrl-sci] (2025)

33. K. Kamala Bharathi, N. R. Kalidindi, C. V. Ramana, Grain size and strain effects on the optical and electrical properties of hafnium oxide nanocrystalline thin films. *J. Appl. Phys.* **108**, 083529 (2010).

34. S. Tsunekawa, K. Ishikawa, Z.-Q. Li, Y. Kawazoe, A. Kasuya, Origin of anomalous lattice expansion in oxide nanoparticles. *Phys. Rev. Lett.* **85**, 3440 (2000), 3443.

35. S. Tsunekawa, S. Ito, T. Mori, K. Ishikawa, Z.-Q. Li, Y. Kawazoe, Critical size and anomalous lattice expansion in nanocrystalline $BaTiO_3$ particles. *Phys. Rev. B* **62**, 3065–3070 (2000).

36. G. Li, J. Boerio-Goates, B. F. Woodfield, L. Li, Evidence of linear lattice expansion and covalency enhancement in rutile TiO_2 nanocrystals. *Appl. Phys. Lett.* **85**, 2059 (2004), 2061.

37. P. M. Diehm, P. Ágoston, K. Albe, Size-dependent lattice expansion in nanoparticles: Reality or anomaly? *Chem. Phys. Chem.* **13**, 2443 (2012), 2454.

38. J. P. Perdew, A. Ruzsinszky, G. I. Csonka, O. A. Vydrov, G. E. Scuseria, L. A. Constantin, X. Zhou, K. Burke, Restoring the density-gradient expansion for exchange in solids and surfaces. *Phys. Rev. Lett.* **100**, 136406 (2008).

39. P. E. Blöchl, Projector augmented-wave method. *Phys. Rev. B* **50**, 17953 (1994), 17979.

40. G. Kresse and J. Furthmüller, Efficient iterative schemes for ab initio total-energy calculations using a plane-wave basis set. *Phys. Rev. B* **54**, 11169–11186 (1996).

41. J. L. Baker, L. H. Jimison, S. Mannsfeld, S. Volkman, S. Yin, V. Subramanian, A. Salleo, A. P. Alivisatos, M. F. Toney, Quantification of thin film crystallographic orientation using x-ray diffraction with an area detector. *Langmuir* **26**, 9146 (2010), 9151.
